# Interprofessional communication with hospitalist and consultant physicians in general internal medicine: a qualitative study

**DOI:** 10.1186/1472-6963-12-437

**Published:** 2012-11-30

**Authors:** Lesley Gotlib Conn, Scott Reeves, Katie Dainty, Chris Kenaszchuk, Merrick Zwarenstein

**Affiliations:** 1 Department of Surgery, St. Michael’s Hospital, 30 Bond St., Toronto, ON M5B 1W8, Canada; 2 Li Ka Shing Knowledge Institute, St. Michael’s Hospital, 30 Bond St., Toronto, ON M5B 1W8, Canada; 3 Center for Innovation in Interprofessional Healthcare Education, University of California, San Francisco, USA; 4 Institute for Clinical Evaluative Sciences and Sunnybrook Research Institute, 2075 Bayview Ave., Room G1 06, Toronto, M4N 3M5, ON, Canada

**Keywords:** Interprofessional care, General internal medicine, Teamwork, Collaboration, Communication

## Abstract

**Background:**

Studies in General Internal Medicine [GIM] settings have shown that optimizing interprofessional communication is important, yet complex and challenging. While the physician is integral to interprofessional work in GIM there are often communication barriers in place that impact perceptions and experiences with the quality and quantity of their communication with other team members. This study aims to understand how team members’ perceptions and experiences with the communication styles and strategies of either hospitalist or consultant physicians in their units influence the quality and effectiveness of interprofessional relations and work.

**Methods:**

A multiple case study methodology was used. Thirty-one semi-structured interviews were conducted with physicians, nurses and other health care providers [e.g. physiotherapist, social worker, etc.] working across 5 interprofessional GIM programs. Questions explored participants’ experiences with communication with all other health care providers in their units, probing for barriers and enablers to effective interprofessional work, as well as the use of communication tools or strategies. Observations in GIM wards were also conducted.

**Results:**

Three main themes emerged from the data: [1] availability for interprofessional communication, [2] relationship-building for effective communication, and [3] physician vs. team-based approaches. Findings suggest a significant contrast in participants’ experiences with the quantity and quality of interprofessional relationships and work when comparing the communication styles and strategies of hospitalist and consultant physicians. Hospitalist staffed GIM units were believed to have more frequent and higher caliber interprofessional communication and collaboration, resulting in more positive experiences among all health care providers in a given unit.

**Conclusions:**

This study helps to improve our understanding of the collaborative environment in GIM, comparing the communication styles and strategies of hospitalist and consultant physicians, as well as the experiences of providers working with them. The implications of this research are globally important for understanding how to create opportunities for physicians and their colleagues to meaningfully and consistently participate in interprofessional communication which has been shown to improve patient, provider, and organizational outcomes.

## Background

Interprofessional communication in general internal medicine [GIM] units is complex. In this setting patient information changes regularly and multiple health care providers [doctors, nurses, physiotherapists, speech pathologists, etc.] simultaneously and interdependently provide daily patient care. Oftentimes health care providers in GIM are changing frequently and so are not working with one another consistently. Previous research on this topic has shown that GIM wards are overloaded with communication ‘genres’ or types that are enacted by varying health care providers at different times to meet their contrasting information needs [1]. Scheduled team rounds and chance hallway encounters, combined with multiple and often duplicate forms of documentation have been shown to result in redundant and ineffective communication strategies which, once recognized, are replaced by new informal “workaround” techniques which further intensify communication challenges [2]. Interruptive communication, which is communicating with other health care providers at ad hoc times, is a regular occurrence in GIM units, as illustrated by observational work conducted in the UK [3]. This research has demonstrated that general medicine physicians and nurses prefer face-to-face communication and electronic message exchanges over others. Qualitative research in the other clinical domains [e.g. emergency department, long term care, and rehabilitation] has similarly found that synchronous communication is the favored mode among most healthcare providers [4-7]. However, it may be the more inefficient one and a factor that contributes to communication failures due to its highly interruptive nature [8-10].

Changes in the way that GIM units are structured and staffed have the potential to transform the culture of communication in these clinical settings. In North America, since the mid 1990s there has been a movement toward hospitalist models of care in GIM, particularly in the United States [11,12], and increasingly in Canada [13-15]. Hospitalists are physicians who focus on the general medical care of hospitalized patients [16]. Hospitalists are typically trained as general practitioners, family physicians, or general internists; in Canada, the majority are family physicians, though some are general internists. Hospitalists are salaried employees of hospitals, with reimbursement agreements varying across the provinces and negotiated therein. All of their clinical work is carried out with hospital inpatients. The increase in the number of hospitalists in the last decade in North America has been attributed, in part, to promising evidence for positive financial and clinical outcomes when hospitalists are present [12], including shorter length of stay and lower hospital costs [17-20]. In academic centers in the United States, restrictions on resident work hours has led to the expansion of hospitalist programs of various design, with factors such as clinical hours worked, compensation structure, and research and administrative activities varying [21].

Recent research in hospital medicine has primarily investigated the effectiveness of hospitalist-staffed medicine wards for achieving these clinical and financial outcomes. There has been less attention paid to the social and cultural aspects, such as health care provider interaction and relationships, related to the transformation to hospitalist units. Despite important research on the cost effectiveness of hospitalist collaboration with nurse practitioners [22,23] and the success of hospitalists’ improved collaborations with pharmacists [24] and case managers [25], little is known about the interprofessional communication in hospitalist staffed settings and how it might differ from that in consultant-based wards. High quality communication has been shown to be an influential aspect of nurse-physician collaboration [26]. Communication is a key activity in patient care as one study of hospitalists’ use of time finds 69% is spent on indirect patient care of which 35% is composed of communication-related activity, i.e., paging other physicians and face-to-face discussions [27].

The present study sought to further our understanding of interprofessional communication on GIM units where either hospitalists or consultant physicians work. Our research question was: how do health care providers in GIM experience communication with one another in a hospitalist versus consultant structured setting? In this article we compare and contrast health care providers’ perceptions and experiences with the quality of communication, interprofessional relationships, and work, based on the communication styles and strategies of hospitalist and consultant physicians on their units. Our analysis considers the influence of the structural design of physicians’ roles in GIM units on interprofessional collaboration experiences.

## Methods

### Study design

A multiple case study methodology was used [28]. The case study approach is useful for exploring social phenomena that are poorly understood in order to generate hypotheses about the phenomena at hand. The research was conducted in five community hospitals in the Greater Toronto Area, in Ontario, Canada. We aimed to achieve some degree of variability across the five settings, yet found much consistency within the GIM units themselves. Three of the five hospitals (H1 – H3) had both consultants and hospitalists. Two hospitals (H4 and H5) had consultants who worked in rotational shifts for one week per month as the on-call emergency department ED/GIM consultant, followed by three or four weeks of strictly outpatient clinics. See Table 1.

**Table 1:** Settings and Interview Participants

**Site**	**Description**	**Interview participants**	**# of units**	**MD staffing model**
Hospital 1	Large community teaching hospital; 5000 staff and physicians	1 Executive Director	6 GIM units	Consultants & Hospitalists
		1 Unit Manager		
		1 Clinical Nurse Educator		
		1 Clinical Nurse Specialist		
		1 Occupational Therapist		
Hospital 2	Acute care community hospital; 3000 staff and physicians	1 Executive Physician	3 GIM units	Consultants & Hospitalists
		1 Consultant Physician		
		1 Unit Manager		
		1 Pharmacist		
		1 Social Worker		
		1 Physiotherapist		
		1 Registered Nurse		
Hospital 3	Large community teaching hospital; 10,000 staff and physicians	1 Consultant Physician	4 GIM units	1 Hospitalist-staffed unit
		1 Pharmacy Director		
		1 Pharmacist		4 Consultant-staffed units
		1 Unit Manager		
		1 Advanced Practice Nurse		
		1 Chief of Medicine		
Hospital 4	Urban community teaching hospital; 3000 staff and physicians	1 Consultant Physician	2 GIM units	Consultants
		1 Unit Manager		
		1 Social Worker		
Hospital 5	Community hospital; 4000 staff and physicians	1 Executive Physician	6 GIM units	Consultants
		1 Executive Nurse		
		1 Consultant Physician		
		1 Unit Manager		
		1 Patient Care Manager		
		1 Dietitian		
		2 Pharmacists		
		1 Nurse Clinician		
		1 Registered Nurse		

The general layout of the GIM units included in this study was similar. In a typical ward, the nursing station was a centrally located space through which all staff passed at some point in their day for charting, telephone calls, face-to-face discussion and computer-based work. Nursing stations were relatively large common areas equipped with chairs and countertops where opportunistic and informal conversations were likely to occur. In addition, wards had designated staff meeting rooms where staff held interprofessional patient rounds and took their breaks. Since patients in GIM are typically elderly with co-morbid conditions they would spend most of their inpatient stay in their rooms, except perhaps when being assessed for mobility by a physiotherapist [PT] in which case they would be walking in the hallway. Health care providers visited patients at different times throughout the day, working independently with the patient, and then reporting back to other team members via documentation or face-to-face discussion. Bedside team rounds were not common in any of these units.

Each of the GIM units across the five settings was staffed by at least one person within the following categories of healthcare professionals: unit manager [typically trained in nursing], registered nurse, social worker, dietitian, pharmacist, PT, occupational therapist [OT], home care coordinator, and resource manager. The majority of unit staff was composed of nurses of varying levels of education and training, either registered nurses, advanced practice nurses or registered practical nurses.

Research ethics approval was obtained from each participating hospital. In order to protect the anonymity of participants, we will not name the hospitals. Consent was obtained from participants in advance.

### Participants

A purposive sample of GIM staff was recruited from each participating hospital. We used a criterion sampling technique [29] to include at minimum one individual from the categories of nursing, medicine, unit management, program executive, and other health care profession (PT, OT, Social Work, etc.) at each site. Thirty-one staff members participated: five physicians, five pharmacists, six unit managers, six nurses, four program executives [two in medicine, two in nursing], two social workers, one dietitian, one PT and one OT. No participants who were approached for an interview declined.

### Data collection

Thirty-one semi-structured interviews were arranged at the convenience of participants. As a result 16 were conducted by telephone and 15 were conducted in-person – depending upon the participants’ preference. The interviews lasted between 20 and 25 minutes. The interview questions explored participants’ experiences with communication with all other health care providers in their units. For instance, questions probed for barriers and enablers to effective interprofessional communication, as well as the use of communication tools or strategies. We sought to elicit participants’ perspectives and opinions about communication generally, as well as profession to profession communication specifically. Interview data was recorded and transcribed. Recruitment of participants stopped when the point of data saturation was reached, that is when no new themes were emerging from the data.

A total of five hours of observation were also carried out, approximately one hour per site. Observations were conducted while shadowing individual interview participants. Observational data were collected following the interviews in order to further explore interprofessional communication patterns, the nature of the GIM context as well as compare the emerging findings from the interview data. For instance, if an interviewee described particular tensions around communication during interprofessional rounds, the observer would shadow that participant during rounds to gain insight to his or her experience therein. If an interviewee perceived the charting room to be a location where the greatest ad hoc interprofessional communication took place, the observer would shadow the participant during charting time in order to explore this. All observations took place on weekdays during formal team rounds or during regular patient care on the wards. The observer was positioned either in a common area such as the nursing station or in meeting rooms during rounds. Observations included staff members who were not directly interviewed for the study but who were made aware of the observations and their purpose in advance. Descriptive observational notes were written by hand and later transcribed into reconstructed reflective field notes by the researcher [30].

### Data analysis

An inductive approach using thematic analysis was used to analyze the data [31,32]. The research team, made up of all authors of this article, came together several times throughout the data collection period. The primary author and data collector (LGC) presented the research team with emerging concepts and categories from the data which were illustrated via coded content from across the interview transcripts and field notes. A thematic framework based on these categories was developed through research team discussion, and was subsequently refined through further data collection and discussion. Team discussion increased the validity of the thematic analysis. All interview transcripts and observational field notes were read iteratively for common themes relating to staff experiences with interprofessional communication in relation to the composition of their units – involving either hospitalist or consultant physicians – and in relation to their roles on the unit – as a hospitalist or consulting physician. At the end of the study period, the research team hosted an interprofessional education workshop for participants and other hospital executives where findings of the qualitative research were presented back for further validation in the form of member checking [33].

## Results

Three main themes related to hospitalist and consultant communication strategies are presented in this section: (1) availability for interprofessional communication, (2) relationship-building for effective communication, and (3) physician vs. team-based approaches.

### Availability for interprofessional communication

Participants revealed that the continuous and reliable presence of hospitalists on the wards created opportunity for both formal and informal interprofessional communication to happen in a timely fashion for patient care. By comparison, staff expressed frustration that consultants who traveled frequently on and off the units were not available to participate in critical decision-making with the rest of the healthcare providers. One participant with experience with both hospitalists and consultants spoke to the frustrations and delays in patient care that resulted from consultants’ persistent absence from the unit during the scheduled weekly interprofessional patient rounds:

*Having the doctor present [at rounds] makes the difference. When you have a doctor on board and you come up with a plan for care you get it validated or changed there on the spot. When there is no doctor, you are creating a plan and then waiting to see the consultant who may veto the plan and you have to re-group and re-present at a later time. When you all have a goal and can see the goal and are working toward the goal, and the attending comes in and says it’s not good, you’re like ‘great, now you need a new goal.’* [Social Worker]

Observations of patient care rounds confirmed that without consultants’ real-time contributions to interprofessional discussion, decision-making and patient discharge were delayed. This was clearly illustrated in this field note excerpt:

A patient was expected to be discharged but is waiting for an EEG [Electroencephalogram] ordered by the consultant. The staff are frustrated that the patient is still here, taking up bed space. The charge nurse calls to the nursing station from the rounds to find out the status of the test. The patient is not scheduled for three days and will have to wait. The charge nurse reports to the others that the consultant in this case doesn’t like to discharge patients without an EEG; she knows this as this scenario has happened before. The PT states that this is “not an ideal world” though and perhaps he should consider discharging the patient and doing it as an outpatient. The consultant is not here to discuss options.

Other field notes indicated that the information provided by and to hospitalists at rounds facilitated interprofessional discussion, discharge planning and other patient care decisions:

Patient 3 is named by the charge nurse and the hospitalist gives the medical update. The hospitalist then turns from the table to the PT sitting on the couch behind her as she describes the patient’s poor mobility. The hospitalist asks the PT what the issue is – is it power, balance, analgesia? The PT replies noting that she doesn’t know what the patient mobility was like before he was admitted. The PT and hospitalist discuss this. The PT says that the patient shouldn’t contemplate going home. The social worker and hospitalist explain that he is planned to go to a nursing home. The PT, social worker and hospitalist then discuss the plan, the patient’s pleasant personality, and cognitive ability.

Participants also reported an overall increase in communication where hospitalists were present during other times of the day when delivering care on the wards. One pharmacist explained that since moving to a hospitalist model in her unit, she had been getting quicker responses to questions and was being approached more often for her opinion creating more effective interprofessional decision-making opportunities. The director of one GIM program, a nurse by training, explained that the advantages of such a model on hospitalist wards were that *“with the hospitalists, the team works together every day. It allows them to work continuously*.” On consultant wards*, “the physician piece is lacking”* she added.

Recognizing the challenges with not being present on the ward, one consultant described an effective communication strategy that relied on the intermediary role of a nurse-clinician, a specialized nurse educator role. In her ward, the nurse-clinician was viewed as a critical link between the consultants and the rest of the health care providers. Without this one human resource, consultants relied on patient information as documented in the chart. According to the explanation provided by this consultant, in the absence of both the nurse-clinician and a clear chart note, patient care was sub-optimal:

*[The nurse-clinician’s] the liaison with nurses, families, social workers, pharmacy. She pulls the team together. There’s not one in every unit which is a pity. In those units it’s hit or miss – we do our best but if people aren’t there then we read the chart. And then if you can’t read the person’s writing, things just don’t get done.* [Consultant physician]

Further, the consultant indicated it was the onus of others to find the consultants when on the ward to deliver or retrieve information, a viewpoint which was consistent with the experience of other participants:

*Collaboration with the allied health people depends on the person. With the social worker it’s good, when they make it their business to talk to us it works. But it’s not ideal because they have to catch us on the fly.* [Consultant physician]

### Relationship-building for effective communication

Participants described their experiences in hospitalist-based units as having positive collaborative relationships and good communication among providers. This included scheduled meetings, such as rounds, and unscheduled discussions in the hallways. The non-physician health care providers contrasted the degree of informality and the existence of social relationships with hospitalists to a lack of any relationship with consultants. Participants on hospitalist wards valued the opportunity to get to know other providers well, both professionally and personally, which they viewed as contributing to a higher level of collaboration and an overall ease with communication:

*Ninety percent of the communication we have is informal. We do have scheduled rounds. We speak to the charge nurse who pools the patient information from the nurses, and she gives us the run down, the highlights. There are also things we do to communicate with the nurses. With allied health it’s on the same note. I’ll approach them on the floor and some will approach us. I know them and their families. I’m fortunate that way.* [Hospitalist physician]

One participant described her less collegial relationships with consultants, reflecting on how this impacted communication generally:

*With the hospitalists, communication is informal. I’m comfortable to approach most of them and am on a first name basis with them. They come out to social events so have fostered the relationship that way. With the consultants it’s much different – there’s no informality, in fact I tend not to speak to them at all.* [OT]

An informal daily “tea time” in one hospitalist ward confirmed that the opportunity to establish interpersonal relationships with other team members was highly valued and the existence of such collegiality fostered dialogue about patient-related concerns. The following observation field note illustrates this well:

*A hospitalist enters the nursing station just as the conversations have ended. “It’s so quiet in here,” he comments. “That’s because we’re working, concentrating really hard,” PT says jokingly. Someone asks why he’s here – it’s not for work but tea. He walks over to the tea and pours himself a cup, “It’s exactly why I’m here,” he says. PT tells the hospitalist that she’s supposed to speak with him about a patient, mentioned at rounds this morning, who she believes walks symptomatic of a Parkinson’s patient. PT offers to show him* [the symptoms] *when she walks the patient. “Will you walk him tomorrow?” the hospitalist asks. PT says yes. “How about we do it together?” the hospitalist suggests. “Whenever you want to do it just come and find me,” he says. PT is satisfied with this. She switches topics, “Did I tell you my good news about winning the provincial* [sport] *tournament?” she asks the hospitalist.*

In another hospitalist ward, an executive-level participant believed the quality of interprofessional relationships influenced the high staff retention rate. As explained to the researcher, the site is not a hospitalist model in total, but “*pieces of Ward B have hospitalists as does the elder care unit.*” According to the participant, *“these staff are able to build relationships with one another over time.”* She added, “*it’s telling that at this site there is not as much turnover with staff which indicates that relationships are probably better.”*

### Physician- vs. team-based approaches

Participants in consultant units characterized them generally as “*physician-centered”* and “*inefficient.”* Some described the dominant and occasionally intimidating position that “*traditional”* consultants in these sites held. There was a reported *“lack of connectedness”* between consultants and other team members, which one participant critiqued stating, “*the physician […] is one part of the team and holds critical and valuable information, but so do other members of the team.”* An interview excerpt with one executive-level physician reflected on this situation explaining, “*the consultants are supreme here. . . [They] have been independent and have not had to cater to management. As a result, the provision of care is not team-based.*”

Several participants provided examples of what they believed to be inappropriate communication with such senior consultants. They attributed this to an older way of thinking about the relations between the professions on the part of said physicians:

*The consultants here are old school mentality. It’s not like in the teaching hospitals where there might be a more distributed or balanced professional hierarchy.* [Unit manager]

A senior executive also explained:

*I have received complaints from nurses that range from “the doctor won’t talk to me” to abusive behavior.* [Senior executive]

Staff across the research sites described some consultants’ communication styles as “*mean and rude*” and that they were generally not responsive to being paged for participation in collaborative decision-making. At one such site, a consultant also reported that communication with PT and OT in particular was “*awful*,” consisting of “*docs writing the order and the PT or OT carrying it out*.” A unit manager wished “*the consultants were more open to learning opportunities for the staff, and not freak out, and be constructive about any problems*.” Patient care and communication with families were believed to be compromised as a result of staff reluctance to subsequently contact consultants who had yelled at them before:

*No one wants to communicate with [consultants] and that does a disservice to our patients. We can’t make plans and communicate what the consultants want to the families. Then, we page the consultants and they get upset that we’re paging them and that no one approached them when they were there.* [Unit manager]

At another consultant site, one staff member expressed a similar reluctance with paging:

*If we are lucky enough to have the consultant at a meeting, he may participate, but we aren’t always so lucky. It is not well supported for us to be able to call them. If they’re there, it’s good for us; otherwise, the first person on the team to see the consultant will relay any messages to him. We can page the consultant but a few of them wouldn’t be happy to get a page to talk about a care plan; for an order clarification it’s fine*. [Social worker]

Another manager described the impact on patient care when consultants’ unprofessional outbursts were witnessed by family members.

*I’ve had to approach a doctor to talk about his language and behavior. I don’t believe it is needed, particularly in front of patients and families. Outbursts might be about tests that weren’t done or lab results that were missed – they’re usually patient-oriented issues like if the patient then has to stay longer or if there was a problem in the emerg. The physicians do care but their communication sucks at times.* [Unit manager]

Finally, our findings suggested that age of consultants was a secondary factor in influencing participants’ experiences with interprofessional communication. For example, a senior nurse felt communication styles had improved among the more junior consultants in one unit:

*[Communication] is better with the newer doctors who treat you more like a colleague. There is always the odd one, however, usually the older ones, who have attitudes that they are above you. I’ve had the occasional time when a doctor has hung up the phone on me. But in the last 10 years the younger doctors have become more respectful and I think that it comes from their training. There are no longer old school ideas where the nurses are treated like the doctors’ maid.* [Nurse]

## Discussion

Our findings illustrate a remarkable contrast in the quantity and quality of interprofessional relationships and work when comparing the communication styles and strategies of hospitalist and consultant physicians in this study. We found that both scheduled and unscheduled face-to-face communication with hospitalists was experienced by staff at all sites to be of an overwhelmingly higher frequency and caliber than that with consultants who some staff did not communicate with in person at all. He continuous presence of hospitalists on the inpatient ward and the perceived and expressed interest of hospitalists to interact in a collegial way with nurses and allied health care providers led to more frequent and effective collaborations in patient assessment and discharge planning. Other health care providers experienced this positively for job satisfaction and morale. Hospitalists themselves promoted the strength of their relationships with other health care providers and believed themselves to be fortunate for having them.

By comparison, nurses and allied health care providers in this study reported a lack of participation and a perceived general lack of interest on the part of consultants in both professional and interpersonal relations reporting poor or non-existent face-to-face communication with them. By and large, consultants were not found to be easily accessible or readily approachable to engage in interprofessional communication. Some health care providers even avoided using real-time communication and relied on charts to convey patient information, a strategy that was recognized as occasionally sub-optimal. Surprisingly, this finding was echoed by consultants interviewed for the research, some of whom were themselves frustrated with the nature of interprofessional communication but felt unable to change the structurally embedded and fragmented way that it happened in their settings. The one-way, task-oriented asynchronous communication described here by a consultant as doctors writing the orders and staff carrying it out resonates with findings from a study of interprofessional rehabilitation units where communication was constrained by hierarchical relations between team members [34]. In this study, junior nurses and support staff were perceived to occupy a lower and less autonomous status than senior nurses, allied health care practitioners, and physicians which impacted the way that others communicated information to them. Our findings suggest that in the GIM settings where consultants primarily work, nurses and allied health care providers work in a silo running parallel to the physician, which can lend to their perception of not being valued in the physicians’ decision making processes.

Sociological research on interprofessional interactions and relations informs us that ward structure and team design can certainly influence the culture of communication and health care providers’ experiences with the quality and effectiveness of interprofessional work [35-39]. Most significant for this study is the interactionist approach, a sociological theory that holds individuals’ interactions with others construct their social world [40]. This provides a useful conceptual framework for understanding how team members’ interactions and communication in their every day work on the unit impact their perceived interprofessional relationships. For instance, research by Ellingson [41], drawing upon Goffman’s backstage-frontstage framework, examines communication on an interprofessional geriatric oncology team. Ellingson conceptualizes the clinic backstage as the space away from patients where providers discuss their assessments, while the clinic frontstage is for patient-provider interactions. Findings from her study highlight the importance of provider information exchange and interactions that happen exclusively in the clinical backstage in order to enact effective teamwork everywhere. In our study in GIM, the hospitalist model of care also helped to establish a cohesive “backstage” clinical environment in which physician involvement in ad hoc, social, interprofessional communication, as well as dialogue about patient care, allowed for the development of collegial relationships with other health care providers and unit members. Indeed hospitalists in this study believed themselves to be fortunate to be able to participate in the health care team in this way. While consultants were perceived by colleagues to be caring and competent doctors, their “frontstage” work alone was viewed to be insufficient for effective team-based care.

Findings from this study also echo previous research about the advantages of health care professionals’ co-location for establishing a sense of “boundedness” suggesting that the closer in proximity providers work to one another, the greater the opportunity and outcome for interprofessional care [42-44]. Hospitalists in our study were perceived to be highly accessible for opportunistic face-to-face communication with other health care providers and also approached others more often for their clinical opinions. In addition, hospitalists’ availability to attend rounds and social-type unit gatherings as a result of not having to leave the ward regularly allowed for more timely and collaborative decision-making regarding patient care, potentially including joint visits to the patient. Baggs and Schmitt’s study [44] of perceptions of collaboration among ICU nurses and resident-physicians similarly found that both availability for and receptivity to collaboration were needed for effective working relationships to occur. This included such traits as openness, respect and trust among colleagues, dimensions of interprofessional interaction that were perceived to be lacking within some consultant-staffed units in our study.

Poor interprofessional communication and relationships in such specialties as surgery and intensive care have been previously attributed to the hierarchical relations that persist between the health care professions [45-47]. Historically, negotiations among health care professionals, especially nurses and physicians, around decision-making, autonomy, and role enactment have been found to result from the hierarchical structure of the medical system which places physicians in a dominant position [38,47-49]. The sometimes-implicit, sometimes-explicit dominance of physicians as compared to other professions in the acute hospital setting has been argued to impede the establishment of collegial communication strategies and relationships between physicians and others [50]; to inhibit some professionals from speaking up even in settings designed for interprofessional interaction [51]; and to reinforce negative interprofessional behavior among junior physicians [52]. While the former findings of poor collegiality and staff inhibition appeared to persist in participants’ experiences on consultant-staffed wards in this study, the latter issue of reinforcing negative interprofessional relations could be changing. The suggestion by some participants in this study that junior consultants communicated differently with their nursing colleagues is indicative of this. The junior consultant-nurse relationship may be making incremental changes responsive to the more complex needs of patients and the system at large. This may be reflective of a redefinition of professionalism in the health care fields with the inclusion of an interprofessional approach [49]. Continuing education in interprofessional collaboration and teamwork skills among physicians of all levels of experience may lead to improved communication in the interprofessional setting. Future research might explore the evolving attitudes and behaviors of this particular group of physicians and its significance for the effectiveness of health care team communication for achieving high quality patient and provider outcomes.

### Study limitations

This study has taken a novel approach to understanding potential transformations in the culture of communication in GIM units by comparing the communication styles and strategies of hospitalist and consultant physicians, as well as the experiences of the health care providers working with them. The study is limited however, first methodologically, and secondly in scope. Methodologically, the study is limited by the empirical generalizability of our findings to GIM units that are structured differently and composed of markedly different health care providers or those with different levels of training and experience. The small sample size is also limited. Our criterion sampling technique aimed to capture a broad range of perspectives and experiences rather than a depth of experience from each category of health care provider. While this was achieved, there remains a gap in our findings about profession-specific understandings and use of communication strategies and techniques in the provision of optimal interprofessional patient care. A longer period of more sustained observations would also help to capture this richer data. In addition, due to the nature of GIM health care providers’ busy schedules, half of our interviews were conducted over the telephone at times that were most convenient for participants and ensured confidentiality, e.g., from home, or off hospital property. A limitation of telephone interviews is the inability to capture non-verbal cues from participants, and perhaps, lack of opportunity to establish a similar rapport between researcher and participant. Future research that consists of all face to face interviews, as well as prolonged observation and additional profession-specific interviewing can address these limitations to further enhance our exploration and understanding of this area.

In terms of the scope of this research, additional interview questions that explore the advantages and disadvantages of physician-staffing models from the patient’s perspective and for communication with health care providers outside of the hospital setting are important perspectives that could not be captured in the present study.

## Conclusions

This study explores communication styles and strategies of hospitalist and consultant physicians in relation to perceptions and experiences of interprofessional relations and work. In many countries, including Canada, the UK and Australia, where interprofessional collaboration is promoted by national and local health care agencies, the knowledge of how interprofessional communication and relations can be meaningfully impacted by hospital staffing models, and their facilitation of effective communication strategies is invaluable to generating a better understanding of the interprofessional approach to care. The hospitalist model may be one approach which helps to place physicians and other health care providers together to engage in mutually advantageous communication, which can result in increased provider satisfaction, collaboration, and high quality patient care.

## Competing interests

The authors declare that they have no competing interests.

## Authors’ contributions

LGC carried out data collection, data analysis and drafted the manuscript. SR contributed to study design, data analysis and helped to draft the manuscript. KD contributed to study design, data analysis and provided critically important intellectual content to the manuscript. CK contributed to study design, data analysis and provided critically important feedback to the manuscript. MZ contributed to study design, data analysis and provided critically important intellectual content to the manuscript. All authors read and approved the final manuscript.

## Pre-publication history

The pre-publication history for this paper can be accessed here:

http://www.biomedcentral.com/1472-6963/12/437/prepub
